# Evolution of manipulative microbial behaviors in the rhizosphere

**DOI:** 10.1111/eva.13333

**Published:** 2022-01-14

**Authors:** Malin Klein, Justin D. Stewart, Stephanie S. Porter, James T. Weedon, E. Toby Kiers

**Affiliations:** ^1^ Department of Ecological Science Vrije Universiteit Amsterdam Amsterdam The Netherlands; ^2^ School of Biological Sciences Washington State University Vancouver Washington USA

**Keywords:** bacteria, conflict, crop improvement, fungi, microbiome, mutualism, soil

## Abstract

The rhizosphere has been called “one of the most complex ecosystems on earth” because it is a hotspot for interactions among millions of microbial cells. Many of these are microbes are also participating in a dynamic interplay with host plant tissues, signaling pathways, and metabolites. Historically, breeders have employed a *plant*‐centric perspective when trying to harness the potential of microbiome‐derived benefits to improve productivity and resilience of economically important plants. This is potentially problematic because: (i) the evolution of the microbes themselves is often ignored, and (ii) it assumes that the fitness of interacting plants and microbes is strictly aligned. In contrast, a *microbe*‐centric perspective recognizes that putatively beneficial microbes are still under selection to increase their own fitness, even if there are costs to the host. This can lead to the evolution of sophisticated, potentially subtle, ways for microbes to manipulate the phenotype of their hosts, as well as other microbes in the rhizosphere. We illustrate this idea with a review of cases where rhizosphere microbes have been demonstrated to directly manipulate host root growth, architecture and exudation, host nutrient uptake systems, and host immunity and defense. We also discuss indirect effects, whereby fitness outcomes for the plant are a consequence of ecological interactions between rhizosphere microbes. If these consequences are positive for the plant, they can potentially be misconstrued as traits that have evolved to promote host growth, even if they are a result of selection for unrelated functions. The ubiquity of both direct microbial manipulation of hosts and context‐dependent, variable indirect effects leads us to argue that an evolutionary perspective on rhizosphere microbial ecology will become increasingly important as we continue to engineer microbial communities for crop production.

## 1 INTRODUCTION

Plants interact with billions of microbes that colonize their roots and rhizosphere. These host‐associated microbiomes—sometimes referred to as the “second genome”—are increasingly identified as a mechanism that allows hosts to expand their genomic and functional repertoire in dealing with a range of ecological challenges (Turner et al., 2013). Such microbiome‐derived capabilities can significantly benefit plant host fitness and performance by providing diverse functions such as mediation of host immunity, increased tolerance to environmental stress, and facilitation of access to novel nutrient sources (Korenblum et al., 2020; Petipas et al., 2021).

Manipulating the untapped potential of the microbiome is a promising research avenue for environmental and agricultural biotechnology—especially in support of sustainable agriculture (Hakim et al., 2021). However, harnessing the microbiome requires an evolutionary perspective on how and why cooperative rhizosphere microbes provide benefits to plant hosts (Friesen et al., 2011). Historically, breeders have employed a *plant*‐centric lens in trying to utilize microbiome‐derived capabilities. One problem with this approach is that the selection pressures driving the evolution of the microbes themselves are often ignored. Rather than being considered as independent actors, microbes are viewed as passive, inanimate accessories used by plants if, and when, needed. This has resulted in the rhizosphere community being considered an “extended root phenotype” (de la Fuente Cantó et al., 2020). From this perspective, microbes are a “tool” that enable hosts to cope with environmental challenges. However, the selection pressures shaping the functional ecology of the microbes themselves are often ignored.

A second problem is the common assumption that microbes are cooperative with hosts because there are direct benefits to a microbial actor of being associated with a healthy host. The problem with this “healthy host equals healthy microbe” model is that it assumes that the fitness of interacting plants and microbes are strictly aligned (Friesen et al., 2011; Kiers et al., 2013) (Box 1). In practice, fitness alignment between different partners is usually difficult to achieve, even in simple communities (Garcia et al., 2019; Minter et al., 2018). Such alignment is usually prevented by the fact that roots are almost always colonized by multiple, competing strains that vary in the net benefits they provide (Burghardt et al., 2018; Kiers & Denison, 2008). This drives an underlying tension because members of the microbiome are each under selection to maximize their benefit from the interaction, leading to conflict and the potential for exploitation (Kramer et al., 2020). As a result, microbes are often under strong selection to defect from costly cooperative behaviors (Denison & Kiers, 2006; Porter & Simms, 2014), especially if they can share in the collective benefits the host provides (e.g., the ~20% of the photosynthetically fixed carbon plants release into the rhizosphere (Stringlis et al., 2018)), while incurring lower costs. Trade‐offs between individual and collective benefits form the basis of this “free rider” problem in the rhizosphere (Denison et al., 2003).

BOX 1 **Evolution of microbial manipulation traits via direct pathways**

Beneficial symbiotic microbes can experience selection to defect from providing benefits to plants if: (i) cooperative traits are costly, and (ii) the benefits are shared equally among competing symbionts. Under these conditions, symbionts are predicted to evolve traits that allow them to exploit the host via multiple distinct pathways, below. Empirical examples of scenarios a, b, and c are rare. This is because fitness data for rhizosphere microbes grown under realistic conditions (i.e., diverse, competing microbial lineages) are extremely limited (but see Burghardt, 2020).

**(a) Privacy.** Traits enabling host manipulation could evolve if the microbial actor is the primary and private beneficiary of host manipulation. In these cases, the benefits of investing in plant manipulation are private and exclusive to the actor and its kin. This could occur if microbial lineages enjoy areas of spatial dominance on the host (Schmidt et al., 2018; Thoen et al., 2019) and this provides unique access to benefits from host manipulation and prevents lineages that do not invest in host manipulation from free‐loading on the benefits of host manipulation without paying their fair share (Chomicki et al., 2020). For example, clonal bacteria that dominate a root section and secrete auxin compounds could be the primary beneficiaries of local ion leakage from plant cells (Talboys et al., 2014). Host deception (a) could also evolve if microbial lineages that invest in host manipulation have a shared special ability to benefit from altering the plant phenotype. Thus, genetic linkage between traits that enable host exploitation and traits that enable benefit from host exploitation can promote the evolution of host manipulation (Magori & Citovsky, 2012).

**(b) By‐products.** Traits enabling host manipulation could evolve if the microbial actor pays no cost to manipulate the host, and thus, alteration of the plant phenotype is a cost‐free by‐product. Microbes excrete waste compounds and secrete extracellular products to maintain homeostasis, defend themselves, communicate with other microbes, and acquire nutrients. If these products also alter plant phenotypes in a way that enables microbes to better exploit the plant (Watt et al., 2006), production of these products can be maintained in microbial populations, even if host manipulation results in public benefits such that other lineages that do not contribute to host manipulation benefit from it. For example, *Pseudomonas* colonies may secrete costly antifungal compounds to kill fungal competitors. As a by‐product, these antifungal compounds could also increase plant resistance to fungal pathogens and protect other microbiome members as well (Denison et al., 2003; Phillips et al., 2004; Yuan et al., 2018) (b).

**(c) Trade.** Traits enabling host manipulation could evolve if the benefits of a microbial actor's costly investment in plant manipulation are a public resource, but the inclusive fitness of the microbial actor is increased due to the actions of other microbes that likewise and collectively benefit from host manipulation. Thus, the microbial actor could cooperatively trade host manipulation with other microbes for other resources such as enzymes, biosurfactants, or metallophores or could benefit more diffusely from cultivating a microbiome community that benefits the actor (c). Such cooperation among distinct lineages in the microbiome could be analogous to cross‐feeding interactions (Fritts et al., 2021; Kramer et al., 2020) that can evolve among cooperating microbes that exchange nutrients.

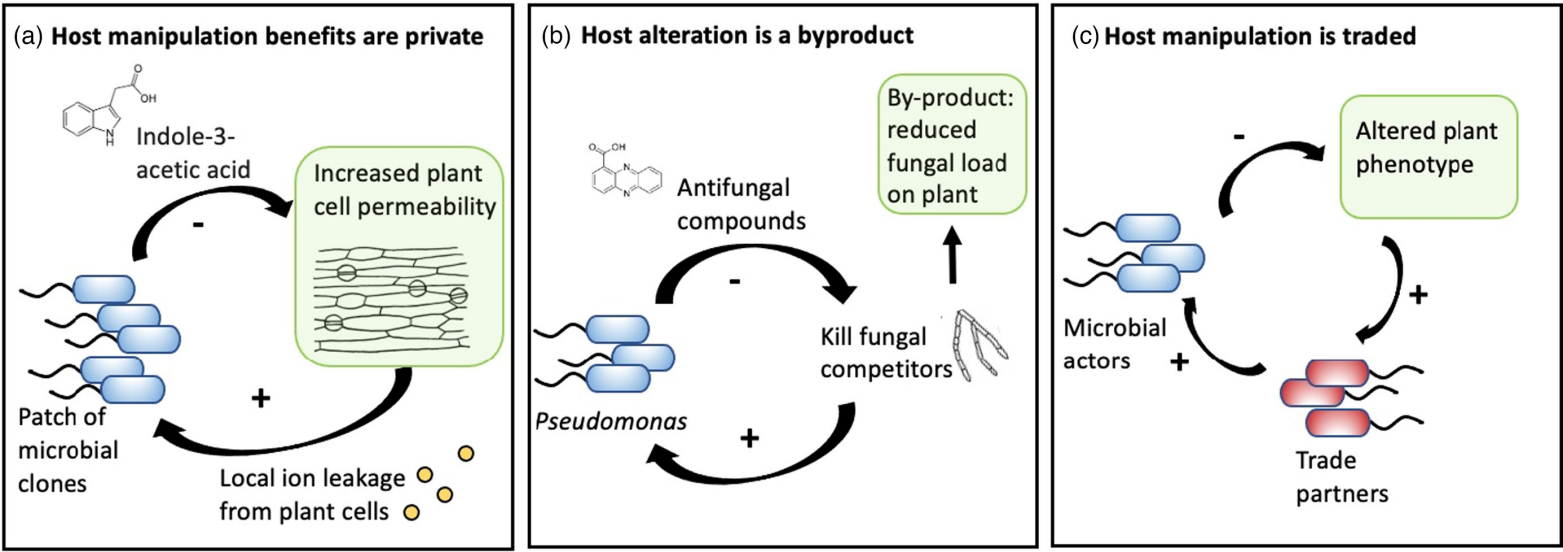

For microbiome members with the potential to confer benefit to the host, there are multiple paths to the evolution of durable host manipulation traits. + indicates benefit; − indicates costs to microbes in the microbiome. Green boxes highlight impacts on plant phenotype.

Plant control of symbiont fitness can select for beneficial microbial symbionts, and this is especially well studied in the legume‐rhizobia symbiosis (Bever, 2015). Here, mechanisms such as pre‐ and postinfection control help to align partners' fitness interests: Legumes can preferentially allocate resources to more beneficial rhizobia both in advance of nitrogen fixation (i.e., “partner choice” (Boivin & Lepetit, 2020; Heath & Tiffin, 2009; Younginger & Friesen, 2019)), and after nodule organogenesis (i.e., “sanctions,” and selective rewarding (Kiers & Denison, 2008; Regus et al., 2017; Westhoek et al., 2021)). However, such fitness feedback is not universal (Oono et al., 2020; Simonsen & Stinchcombe, 2014), and some rhizobial lineages have evolved to escape such feedback (Price et al., 2015). Benefits to a plant can also evolve as a by‐product of selection on symbionts. Here, genetic linkage between private (i.e., selfish traits) and cooperative benefits can help explain how cooperative behaviors are maintained in microbiome members (e.g., Foster et al., 2004; Li, de Jonge, et al., 2021). For example, an initially harmful rhizosphere bacterium, *Pseudomonas protegens*, can rapidly evolve into a plant mutualist. *Pseudomonas protegens* mutants that reduce secretion of costly phytotoxins may both become competitively superior to other strains in the rhizosphere and confer higher benefit to the host plant (Li, de Jonge, et al., 2021).

More generally, our current *plant*‐centric lens has led to great breakthroughs in understanding how plant hosts manipulate the microbiome to their advantage (Pascale et al., 2020). However, it fails to recognize that a *microb*e‐centric view is equally important. Microbes are under selection to increase their own fitness, even if there are costs to the host. As a result, cooperative microbes often evolve sophisticated, potentially subtle, ways to manipulate and modify the phenotype of their hosts, and other microbes in the rhizosphere. These strategies can help them gain extra resources. These manipulative interactions are often overlooked in cooperative plant–microbe associations. However, an evolutionary understanding of these dynamics will become increasingly important as we attempt to engineer the microbiome to increase the benefits microbes confer to plants in cropping systems (Denison, 2019; Li, de Jonge, et al., 2021). For example, if certain microbiome compositions are better at promoting host fitness, and these benefits are shared across the community because of microbiome inheritance or host preference, then this can be used to help engineer certain communities over time (e.g., Wippel et al., 2021).

Our aim is to explore how nonpathogenic (i.e., putatively cooperative) microbes in the rhizosphere and root endosphere can (indirectly) manipulate the phenotypes of their hosts to their own advantage. Here, host manipulations are defined as alterations by the microbe to the host that benefit the microbe, regardless of a positive, negative, or neutral outcome for the host. Our examples span the full extent of the plant–soil interface: We cover microbes living inside, outside, or between plant cells, in the rhizoplane (root surface), as well as inhabitants of the larger rhizosphere (Barriuso et al., 2008). We discuss endosymbionts, like N_2_‐fixing bacteria and symbiotic mycorrhizal fungi, as well as plant growth‐promoting rhizobacteria (PGPR), also known as plant health‐promoting bacteria (PHPB). Our goal is not to review the diversity of benefits conferred by root‐associated microbes, but rather to offer an evolutionary perspective that highlights: (i) the ways in which rhizosphere microbes use manipulative behaviors to increase their own fitness, and (ii) how this can affect the phenotypes of crop plants.

Our discussion begins with a review of direct effects, whereby putatively cooperative microbes have evolved mechanisms to directly manipulate the host to the microbes' advantage. We then move on to indirect effects, whereby the microbial actors manipulate each other, and these interactions can be either positive or negative for the host depending on biotic and abiotic conditions. If these consequences are positive for the plant, they can be misconstrued as traits that have evolved to promote host growth by researchers, even though they are merely indirect side effects of microbial traits that have evolved due to selection for other functions. By examining the evolutionary context of ecological interactions between host and microbe in the rhizosphere, as well as among different microbial community members, we can better understand how these interactions can be harnessed to increase crop productivity and resilience.

## 2 DIRECT MANIPULATION

### 2.1 Modification of root growth, architecture, and exudation

**FIGURE 1:**
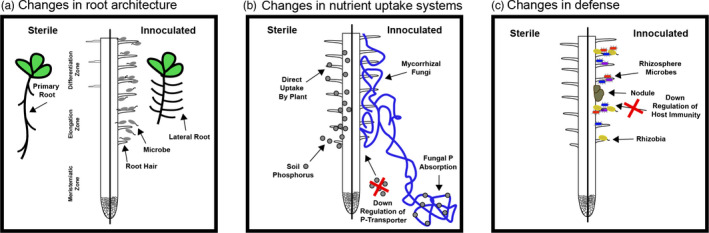
Phenotypes can be misconceived as “positive” for the host plant, but instead favor the growth and success of the microbial actor at a cost to the host plant. (a) Changes in root architecture: Microbes are able to alter the three‐dimensional architecture of plant roots traits including length, branching, and root hair density via microbial production of plant hormones (e.g., IAA) or by modifying existing root exudates in the rhizosphere. Through manipulation, the microbes may gain more physical root space to live on, while the plant may lose access to resources such as water deep in soils. (b) Changes in nutrient uptake systems: Restriction of plant host nutrient acquisition mechanisms creates a dependence on the microbe for critical plant nutrients. A prominent example of this is with mycorrhizal fungi that can downregulate plant P‐transporters that creates a dependence on the fungi by the plant for soil P resources. (c) Changes in defense: Rhizosphere microbes can change plant defenses to benefit microbial actors. This manipulation strategy has been successful for across the prokaryotic and eukaryotic kingdom as it is seen in both fungi and bacteria. When this occurs potentially pathogenic microbes could gain a relative advantage and negatively influence plant health

The most direct and easily observable influence of rhizosphere microbes involves modification of the growth and physiology of host‐plant roots (Figure 1a, Table 1). This can occur via microbe‐induced changes to root architecture, overall root growth, or the amount, composition and spatial dynamics of root exudation. By taking advantage of the high degree of plasticity in root growth, rhizosphere microbes can manipulate the three‐dimensional architecture of root systems, including changes in length, lateral root branching/elongation, and root hair densities (Fan et al., 2011; Vacheron et al., 2013).

**TABLE 1:** Summary of the types of manipulation by microbes reviewed in this article

Manipulation	Type of effect	Example and citation
Root Architecture	Direct	Microbes living on and inside plant roots can elicit root exudation (Dobbelaere et al., 1999; Idris et al., 2007) as well as degrade plant hormones (Glick, 2005; Spaepen et al., 2007). This directly alters the three‐dimensional shape of roots in soils (Fan et al., 2011; Vacheron et al., 2013).
Root Exudates	Direct	Microbes increase rhizodeposition (especially amino acids) by the host (Kudoyarova et al., 2014; Talboys et al., 2014).
Nutrient uptake	Direct	Colonization of plant roots by arbuscular mycorrhizal fungi and subsequent downregulation of nutrient transporters restricts the ability of the host to directly take up P (Burleigh, 2001; Pearson & Jakobsen, 1993; Ravnskov & Jakobsen, 1995; Smith et al., 2003, 2004). Some rhizobia can force nodulation (Ratu et al., 2021), or co‐invade nodules along with other strains despite being ineffective N_2_‐fixers (Porter et al., 2019), while escaping the host control (Pan & Wang, 2017; Price et al., 2015). Rhizobia accumulate carbon at cost of nitrogen fixation (Oono et al., 2009; Ratcliff et al., 2008). This leads to an overall reduction of the hosts fitness.
Immunity & defenses	Direct	Microbes suppress the plant host immune response to establish symbiosis at the expense of the host's defense against “true” pathogens (Frew et al., 2018; Wood et al., 2018). Microbes alter the hosts rhizodeposition to chemotactically attract same‐species “reinforcement” (Ray et al., 2018).
Interspecific Competition	Indirect	Competition between microbial species may alter the membership of the soil microbiome. Potentially, beneficial bacteria may be removed from populations and thus cannot colonize plant roots, that is, antibiotic and antifungal production (Cook et al., 1995; Weller et al., 2002; Yuan et al., 2018). Host plants benefit from hyperparasitism (Inayati et al., 2020), or from chemical homeostasis (Finkel et al., 2020), that is, inhibition of (unfavorable) growth stimulated by other bacteria.
Interspecific facilitation	Indirect	Rhizosphere microbes facilitate each other's colonization, externally and intracellularly (De Jaeger et al., 2010; Harman et al., 2004; Poveda et al., 2019; Taktek et al., 2015)
Nested Interactions	Indirect	Bacterial endosymbionts of fungi can modulate fungal pathogenicity as well as carry a metabolic cost for the fungal host. Energy diverted to maintaining a bacterial endosymbiont could lessen potential benefits conferred to a plant by a fungal partner (Alabid et al., 2019; Uehling et al., 2017).

Note

For each form of manipulation, the type of effect on the plant host and a brief example are given.

Although PGPR produce a range of compounds that can influence root growth, some of the most common examples of architectural manipulation involve rhizobacteria species that secrete indole acetic acid (IAA), which increases root production and can have effects on host hormonal signaling processes (Dobbelaere et al., 1999; Idris et al., 2007), such as that for auxin. Bacteria can manipulate root architecture by producing these hormones directly or alternatively by perturbing concentrations and/or the transport of hormones within the host itself (Glick, 2005; Spaepen et al., 2007). It is generally assumed that the resulting changes to root architecture increase the efficacy and efficiency of belowground nutrient acquisition for hosts (Freschet et al., 2021) (as detailed below) and are thus an evolutionary advantage to host plants. This is not consistently true: Microbe‐induced architectural changes do not always directly benefit hosts (Denison, 2019), but they can benefit the microbe performing the manipulative behavior (see Box 1). Increases in total root production have largely been considered beneficial because increased root growth has the *potential* to increase a host's ability to forage for nutrients—a desirable trait for crop production (Jung et al., 2019). However, stimulation of root growth can be costly and involve trade‐offs in other traits (Denison, 2019), such as P uptake ability (Talboys et al., 2014). For example, many *Bacillus* species are beneficial members of the plant microbiome (Blake et al., 2021). In wheat, however, the presence of the rhizobacterium *Bacillus amyloliquefaciens* increased root growth, but a Pi transporter of the host was simultaneously reduced, resulting in a decrease in Pi uptake in low P soils (Talboys et al., 2014). Similarly, inoculation of *Arabidopsis thaliana* with *B*. *megaterium* resulted in inhibition of the primary‐root growth of hosts, and an increase in number and length of lateral roots (López‐Bucio et al., 2007). The enhancement of lateral branching in roots by microbe‐produced auxins can have negative consequences for hosts because they inhibit crucial root elongation needed to, for example, extract water in deeper soil layers (Grover et al., 2021). In contrast, microbes may benefit from the provision of increased suitable root habitat (Watt et al., 2006). Such host root manipulation will be under even stronger selection if the microbial actor producing the trait directly benefits from the extra space. This creates a clear conflict of interest between root phenotypes that favor host fitness versus microbial fitness. As the number of different types of symbioses increases (e.g., mycorrhizae and N_2_‐fixing bacteria), these dynamics may also change (Afkhami et al., 2021).

The production of root‐manipulating substances by rhizosphere microbes can also increase microbial access to host metabolites. Studies of wheat cells (i.e., *Triticum aestivum* callus cultures) show higher membrane permeability and ion leakage from cells treated with auxin compounds (e.g., IAA (Filek et al., 2004)), suggesting that rhizobacteria may directly (and rapidly) increase the “leakiness” of cells, but at the expense of Pi uptake by the roots (Talboys et al., 2014). This is a manipulation trait that is likely to spread through a rhizosphere population because of the direct benefits potentially received by the manipulator (Box 1). A similar example is the production by *Bacillus subtilis* of cytokinins that stimulate rhizodeposition by the host, specifically increasing root exudation of amino acids. The exuded amino acids directly stimulate the growth and population density of *B*. *subtilis* with no apparent advantage for the host, but at the cost of amino acid production for the host (Kudoyarova et al., 2014; Mäder et al., 2002).

Apart from auxins, it is also well documented that phenazine and zearalenone produced by microbes can trigger increased amino acid efflux by plant roots. However, the required concentrations are relatively high, leading to the question of whether carbon gains for microbes are above carbon costs incurred to produce these manipulative compounds (Phillips et al., 2004). Past work in crop plants found that active influx rates of amino acids to plant roots can exceed passive efflux rates by over 500% when microorganisms are absent (Phillips et al., 2004). The passive efflux of amino acids is mainly driven by concentration differences inside and outside root cells (10 mM versus 0.1–10 μM). If microbes were present, they would potentially consume the exuded amino acids, thus creating a sink and cause a continuous passive efflux of amino acids. Additionally, several microbial products—including those produced by soil microorganisms such as *Pseudomonas* and *Fusarium*—consistently lead to increased exudation of amino acids from roots that can then be consumed by microbes (Phillips et al., 2004). Manipulative studies have shown that the application of long‐chain fatty acids and amino acids to host roots tends to recruit *Pseudomonas* strains that can help host plants resist foliar pathogen infection, which can be considered a direct benefit to the host (Wen et al., 2021). However, the cost to the plant of producing these compounds themselves is rarely measured. Given that the benefits of these *Pseudomonas* strains to hosts may only be realized by “future” plant generations grown in the same soil (i.e., a soil legacy effect, see section on indirect effects below), the cost of producing these compounds by the host may outweigh any immediate direct benefit to the plant, regardless of whether the exudates function as a public good for subsequent generations (Denison et al., 2003).

Induction of root exudates can be so tightly linked to the root microbiome that the process is termed SIREM (Systemically Induced Root Exudation of Metabolites). This concept posits that microbial communities induce specific systemic changes in host root exudation such that even small changes in the microbial community can lead to large alterations of host phenotypes (Brinker et al., 2019; Korenblum et al., 2020). However, an open question is how specific root exudation profiles benefit specific microbial strains. For a costly microbial behavior to evolve, individuals (or clone mates) performing that behavior must receive a direct benefit (Denison et al., 2003) (see Box 1). However, measuring direct benefits of specific compounds to competing microbial strains is difficult, and research linking root exudation to fitness data of strains within microbial communities is rare. If specific resources, such as root exudates, could be preferentially allocated/directed to the microbes driving their release, this could provide a direct benefit to microbes. Evidence for this idea comes from the study of Korenblum et al. that monitored the spatial distribution of specific metabolites across parts of the root system, showing the distinct regions (including tips of lateral roots) of metabolite accumulation (Korenblum et al., 2020). This could suggest that the microbes are successful at manipulating the spatial arrangement of the resources to their benefit. Future work could aim to link spatial arrangements of metabolites with Green Florescent Protein (GFP)‐labeled strains of microbial strains to test whether their exudation directly benefits the microbial actors performing the manipulation.

The studies reviewed in this section lead to the question of if (and when) breeders should select for increased rhizodeposition or microbe‐induced architectural changes of crop plants. It is still an open question whether selecting for these microbe‐mediated traits can lead to major improvements in crop performance. Natural selection has tested various solutions to modulating carbon allocation below ground (Denison, 2019). Therefore, breeding simply to attract more feedback with PGPR is unlikely to lead to increases in host performance unless the scope for conflict between plant and microbes is somehow further reduced. Several experimental evolution approaches have elegantly shown how this conflict can be reduced when a single host genotype interacts with a single microbial genotype, over relatively few generations (Batstone et al., 2020; Li, de Jonge, et al., 2021; Quides et al., 2021). For example, the plant‐antagonistic bacterium *Pseudomonas protegens* can transition to a rhizosphere mutualist of *Arabidopsis thaliana* within 6 plant generations and increase fitness via improved competitiveness for root exudates and enhanced tolerance to the plant‐secreted antimicrobial compound scopoletin (Li, de Jonge, et al., 2021). *Ensifer meliloti* rapidly adapts to its local *Medicago truncatula* host genotype via mutations in putative signaling genes on a symbiosis plasmid (Batstone et al., 2020). Furthermore, plant traits can be genetically altered to attract and influence different microbiome partners with great precision (Geddes et al., 2019). These approaches should now be expanded to more complex communities, spanning different types of microbially induced host manipulations.

### 2.2 Manipulation of nutritional symbioses

Plants have evolved mechanisms to increase their nutritional advantage by favoring the growth of specific rhizosphere members (Denison & Kiers, 2011). It is less well understood, however, how microbes manipulate the nutritional uptake of plants to their advantage (see Table 1). One idea is that putatively cooperative rhizosphere microbes may be able to gain fitness benefits by actively restricting their partners' ability to obtain resources directly from the environment. Theoretically, if a microbe can manipulate a host's autonomy (i.e., such that the host needs the microbe for basic functions like obtaining nutrients), this should drive the evolution of dependence and increase the potential for more resources being directed to the microbe (Figure 1b). As hosts become more reliant on their microbes to supply resources, the value of those resources increases (Wyatt et al., 2016). This can even create an addiction dynamic, whereby host autonomy is lost as reliance on microbe‐derived nutrients increases (Fisher et al., 2017; Sullivan, 2017). This can have both negative and positive consequences for the host, depending on the context, stressing the need to consider net benefits when studying these types of interactions (Keeling & McCutcheon, 2017).

The most well‐studied rhizosphere example relates to traits that enable mycorrhizal fungi to restrict the ability of their plant partners to directly take up nutrient resources from the soil (Burleigh, 2001; Pearson & Jakobsen, 1993; Ravnskov & Jakobsen, 1995; Smith, Smith, & Jakobsen, 2003, 2004) (Table 1). A root colonized by AM fungi has two pathways through which P can be absorbed: (i) a direct pathway through root cells and root hairs and (ii) a fungal pathway, in which P is absorbed by fungal mycelium and translocated to fungus/plant interfaces (Jansa et al., 2005). By restricting the direct uptake pathway, a fungus can potentially increase the amount of carbon gained from trading phosphorus (Werner et al., 2014) (Figure 1b).

Mechanistically, the colonization of plant roots by arbuscular mycorrhizal fungi has been shown to restrict the ability of hosts to directly take up P, while driving the downregulation of nutrient transporter genes (e.g., P, N, Zn) (Burleigh, 2001; Pearson & Jakobsen, 1993; Ravnskov & Jakobsen, 1995; Smith et al., 2003, 2004). For example, in *Medicago truncatula*, colonization of mycorrhizal fungi was associated with a downregulation in high‐affinity phosphate transporters (e.g., MtPt2), and even putative nitrate transporters (Burleigh, 2001). In some cases, downregulation is so severe that AM fungal colonization was associated with a complete inactivation of the direct P uptake pathway via both root hairs and epidermis (Smith et al., 2004).

This downregulation can be explained by conflicting selection pressures on host and symbiont: (i) If maintaining the direct uptake pathway is costly when the fungal pathway is providing less‐costly P, then plants will experience selection to downregulate the direct uptake pathway; (ii) if restricting the direct uptake pathway makes P more valuable, leading to a higher C:P exchange ratio, the fungus will be under strong selection to evolve ways to manipulate the host's nutrient uptake system to downregulate the direct uptake pathway. In these two scenarios, the fitness interests of the two partners are only superficially aligned. In scenario (i) where P from the direct uptake pathway becomes less‐costly compared to the fungal pathway (i.e., high local P availability), plant hosts will be under selection to increase their direct uptake, while fungal symbionts will still be under selection to downregulate this pathway (Bever, 2015).

Hijacking of nutrient pathways has been explored theoretically (Wyatt et al., 2016), but more data are needed to confirm when and how it operates in living systems. The contribution of the mycorrhizal pathway to total plant P uptake differs across conditions, including fungal partner identity, nutrient availability, and host health (Ngo et al., 2021), making it difficult to find the exact conditions when nutrient pathway hijacking is likely to operate. In addition, an open question is whether such a trait can be an evolutionarily stable strategy for the fungus, given that multiple strains of competing AM fungi on a single root system may benefit from the “public good” of restricting the host's direct uptake pathway. Another option is if suppression of P uptake by AM fungi is cost‐free, in which case the benefits of plant dependency are simply a by‐product (Box 1b). It is clear that the capacity to downregulate host nutrient transporters varies widely among AM fungi, as does the range of nutrient conditions under which this strategy is effective (Burleigh, 2001). This suggests that the trait may be costly under certain conditions. Theoretical modeling predicts that restriction is most likely to evolve under conditions when the act of restricting is under the control of a single agent (e.g., a single fungus (Wyatt et al., 2016)).

The ultimate fitness consequences of P uptake restriction for plants are not known. Is microbe‐directed restriction costly to the host only under very high nutrient availability, such as in high‐input agricultural fields (Johnson & Gibson, 2021)? In many cases, symbiotic associations are plastic in response to external conditions, such that colonization is drastically reduced when phosphorus availability is high (Balzergue et al., 2013; Müller & Harrison, 2019). When nutrient availability remains consistently very high, it can become a selection pressure that has been proposed to drive some plant lineages (e.g., Brassicaceae) to lose the ability to interact with mycorrhizal fungi completely (Werner et al., 2018). This raises the possibility that hijacking, or some other selection pressure from interacting with AM fungi, was so costly in the past that some plants totally abandoned—rather than modulated—the symbiosis (Werner et al., 2018). A key future question is whether the intake efficiency of the two pathways is similar. If so, then the plant is effectively moving in a flat fitness landscape. If the efficiency differs, however, then we expect costs or benefits from relying on fungi. Understanding ancestral plant states for nutrient extraction mechanisms could help answer this question. For example, it is important to determine whether plants have ever been able to take up complex forms of phosphorus in the absence of fungal symbionts.

Aside from fungal symbionts, plants also enter into intimate (and potentially costly) endosymbiosis with N_2_‐fixing bacteria (Bailly et al., 2006; Sachs et al., 2018). Lineages of Proteobacteria—broadly known as rhizobia—have evolved diverse strategies to trigger the formation of nodules on legume roots. Although N_2_ fixation is costly for rhizobia, they also reap a massive fitness increase: Nodules are often founded by a single rhizobium cell, yet can release thousands to millions of rhizobium progeny upon nodule senescence (Müller et al., 2001; Porter & Simms, 2014). Rhizobia are intracellular symbionts that consume a great deal of legume resources—often 4%–14% of recently fixed photosynthetic carbon (Kaschuk et al., 2009). Although many legumes are capable of preferentially initiating nodulation with (Heath & Tiffin, 2009; Regus et al., 2014; Younginger & Friesen, 2019), and allocating resources to superior strains of their rhizobial partners (Oono et al., 2020; Westhoek et al., 2017, 2021), that provide optimal benefits, these defenses are imperfect. Rhizobia that provide low or no benefit to the host succeed in infecting nodules in many interactions (Gano‐Cohen et al., 2016; Porter et al., 2019; Simonsen & Stinchcombe, 2014). In fact, rhizobia have evolved multiple tactics for manipulating legume hosts to their own advantage, including (i) during nodule initiation and (ii) during resource exchange (see Table 1).

To initiate a nodule, a legume species often requires rhizobia to respond to the particular cocktail of flavonoids it secretes from its roots by producing the particular suite of Nodulation (Nod) factors detected by the plant's LysM receptors (Zipfel & Oldroyd, 2017). However, some rhizobia manipulate their host plants by bypassing this requirement and forcing nodulation in the absence of Nod factor signaling. To do so, they can use effector proteins similar to those of pathogens to hijack the host symbiosis signaling cascade to promote infection through cracks in the plant epidermis or other intercellular spaces (Okazaki et al., 2013, 2016; Sachs et al., 2018; Teulet et al., 2019). For example, some *Bradyrhizobia* inject pathogenic‐like effectors to induce nodule formation, even in soybean mutants defective in Nod factor perception (Ratu et al., 2021). These effectors can work to sidestep the Nod factor signaling pathways, though in some cases legume resistance alleles allow the plant to terminate such effector‐triggered nodules (Tang et al., 2016; Yasuda et al., 2016). Ineffective rhizobia can also infect legume hosts as stowaways (Figure 1c). Nonbeneficial strains that lack the genes to initiate nodules or fix nitrogen can co‐invade nodules with wild‐type strains, where they enjoy access to diverse resources in the nodule and can reduce legume fitness (Gano‐Cohen et al., 2016; Porter et al., 2019). Even though legumes often rely on specific signals as an indication of partner compatibility, mutations that reduce or abolish a strain's ability to fix nitrogen are often invisible to hosts (Westhoek et al., 2017; Younginger & Friesen, 2019). The result of these various vulnerabilities is the persistence of rhizobium strains that are highly promiscuous despite conferring low benefits. *Sinorhizobium fredii* NGR 234, for example, can infect 112 different legume genera and is often an ineffective symbiont (Krysciak et al., 2015). Lastly, gibberellin production by rhizobia in already formed nodules can result in a reduction of subsequent nodulation, potentially overriding the host's own ability to regulate nodule numbers, and reducing the number of potential competitors for rhizobia (Tatsukami et al., 2016).

Once nodulation has occurred, rhizobia have also evolved ways to manipulate legumes during resource exchange. During resource exchange, both host and symbiont will be under selection to maximize their benefits while minimizing costs (Denison & Kiers, 2006; Porter & Simms, 2014; Sachs et al., 2018; Westhoek et al., 2021). This tension has led to the evolution of diverse rhizobium strategies to contribute less nitrogen to the host while hoarding more resources for their own progeny (Sachs et al., 2018). For example, during the course of nodulation, legumes in the Inverted Repeat‐Lacking Clade (IRLC) force rhizobia to terminally differentiate into highly efficient nitrogen‐fixing organelle‐like bacteroids (Oono et al., 2009). To do so, these legumes can inundate rhizobia with nodule cysteine‐rich (NCR) peptides, some of which induce sub‐lethal toxicity, which can curtail rhizobium reproductive potential (Pan & Wang, 2017; Price et al., 2015). However, some rhizobium strains have evolved a peptidase, Hrrp, that specifically degrades NCR peptides that can allow the bacteria to escape host control and proliferate rapidly without conferring much nitrogen to the host in both novel (Pan & Wang, 2017; Price et al., 2015) and co‐evolved symbiotic interactions (Wendlandt et al., 2021). Such nonfixing rhizobia can impose large costs on hosts, potentially resulting in a 12–28% reduction in leaf nitrogen content and an ongoing evolutionary arms race between symbionts and hosts (Regus et al., 2015).

Rhizobia can also hoard resources at a cost to their host. Some rhizobia can accumulate carbon in the form of the storage molecule poly‐3‐hydroxybutyrate (PHB), although this comes at the cost of using carbon to fix nitrogen for the host. PHB reserves can support rhizobial survival (Ratcliff et al., 2008) and even reproduction in the absence of an external carbon source. This means that rhizobia face a trade‐off between allocating carbon to nitrogen fixation or toward improving the survival of their own progeny. Mutant rhizobia incapable of synthesizing PHB fix more nitrogen on legume than strains capable of synthesizing PHB (Cevallos et al., 1996). While not all rhizobia employ this tactic (e.g., differentiated rhizobia), there is evidence that some lineages of rhizobia may exploit legumes by accumulating PHB instead of fixing nitrogen (Oono et al., 2009).

### 2.3 Manipulation of host immunity and defenses

Microbes can directly manipulate host defenses—restricting or boosting immunity according to their own fitness requirements. Basic suppression of host innate immunity is a requirement for the establishment of endosymbiotic root mutualists, like mycorrhizal fungi and rhizobia, to prevent the host's cells from recognizing and countering the microbes (Zipfel & Oldroyd, 2017). Suppression of host immunity is therefore directly linked to the fitness of the microbial actor and plays a role in both pathogenic and mutualism dynamics (Li et al., 2021) (Figure 1c, Table 1). The basic blueprint of mycorrhizal signaling for the manipulation of host innate immunity has proved to be so versatile that rhizobia could adopt components of it, resulting in a shared symbiosis signaling pathway (Oldroyd, 2013).

While terms like intracellular “accommodation” are often used to stress the *plant host* role in colonization (Saeki, 2011), microbial manipulation of the host's immunity underlies these processes. Recent work has stressed the fine balance between immunity versus symbiosis signaling, and the role of microbe‐associated molecular patterns (MAMPs) in shifting that balance (Newman et al., 2013). For example, the perception of pathogen‐derived signals and symbiosis‐promoting factors involves a shared coreceptor in crops, such as rice. In these cases, rice mutants missing this co‐factor show reduced arbuscular mycorrhizal symbiosis (Miyata et al., 2014).

From a host perspective, distinguishing mutualists from pathogens often requires finely tuned signaling systems and can even be based on receptor competition that facilitates a switch from defense to symbiotic signals (Zhang et al., 2021). Likewise, transcriptomic analyses have shown that many defense‐related genes are upregulated in the legume hosts during the early stage of the legume–rhizobia interaction, but the majority are subsequently suppressed to allow for successful colonization (Kouchi et al., 2004). More generally, interactions with symbiotic microbes can drive host plants to generate a transient, weak, defense response, similar to that found in plant–pathogen interactions, and play a key role in the successful spread of rhizosphere microbes.

While downregulation of host defense facilitates the internal accommodation of microbes, this can come at a cost of potentially reduced defense against “true” pathogens and parasites. This has been shown in the N_2_‐fixing symbiosis: Legume genotypes that form higher numbers of nodules tend to be more vulnerable to parasitism by gall‐forming nematodes (Wood et al., 2018). Likewise, in two different varieties of wheat (*Triticum aestivum*), colonization by four species of AM fungi also suppressed the host's ability to defend itself against plant–parasitic nematodes (Frew et al., 2018). The researchers found that interactions with AM fungi resulted in decreased concentrations of the benzoxazinoid glucoside defense compounds, which coincided with increases in the population size of the nematode *Pratylenchus neglectus* by between 47% and 117%. While fungal colonization was associated with nutritional increases of phosphorus, potassium, and zinc for the host root, overall plant biomass was still reduced by 24%, indicating a potential fitness cost for the host.

Like AM fungi and rhizobia, endophytic bacteria can also regulate host defenses, but the direct benefit experienced by members of this guild is less clear. *Alcaligenes faecali*s has been shown to influence the phenolic root exudate profiles of colonized okra plants. This has been called a process of “self‐fortification” for its presumed role in protecting the host against pathogens (Ray et al., 2018). Phenolics in the okra root exudates chemotactically attract *A*. *faecalis*, which then colonizes the rhizoplane and further alters the phenolics composition of host's root exudates to aid its own survival. More work is needed to determine if/how the endophyte uses host carbon to defend its own physical space in the rhizoplane against competitors, and whether this interaction has the potential to turn pathogenic as context changes (e.g., under low pathogen presence so that *A*. *faecalis* does not have to defend its physical space).

## 3 INDIRECT MANIPULATION

Rhizosphere microbes can evolve traits that allow them to manipulate plants directly, but they can also evolve traits, in which plant manipulation is an indirect consequence of their interactions with other microbes. In these cases, the host root is the site of manipulation, which takes place among the microbial actors themselves. If these consequences are positive for the plant, they can be misconceived as “promoting” host growth, even though they are merely indirect side effects of interactions subject to evolutionary processes that do not directly involve the plant (Box 1b). Such by‐product effects are common in the rhizosphere, but may be less‐consistently beneficial (Denison, 2019). In the context of selecting beneficial microbes for crop growth, the difference between direct and indirect manipulation therefore becomes crucial. This is because: (i) the consequences of microbe–microbe interactions for the host plant may be strongly dependent on context, such that small changes in the environment shift host outcomes from positive to negative, and (ii) co‐evolutionary dynamics in microbe–microbe interactions that benefit hosts may be harder to consistently harness in cropping systems.

Understanding the evolution of ecological interactions among rhizosphere microbes can help develop blueprints for how they can be manipulated. For example, when one rhizosphere microbe outcompetes other microbes, the outcome will only be positive for the plant if the winner consistently acts as a mutualist. The root‐associated fungal endophyte of the *Colletotrichum gloeosporioides* clade suppresses the growth of both its close and more distant pathogenic relatives in the root systems of *A*. *thaliana*. The endophyte restricts pathogen growth at an early infection stage and slows its reproduction *in planta* by outcompeting it. Under *in vitro* conditions, however, growth and reproduction of the pathogen are not suppressed by the endophyte. This suggests that the observed benefits *in planta* are a side effect of direct fungal warfare to compete for space and resources inside the root (Box 1b). Pathogen protection for the plant is a beneficial, but dynamic and context‐dependent, by‐product. This context dependency is even more important in changing nutrient conditions: Under phosphate‐deficient conditions, the previously beneficial endophyte becomes pathogenic (Pathompitaknukul et al., 2020).

### 3.1 Microbial warfare on and within host roots

Because rhizosphere microbes live in dense communities, there is usually intense multispecies competition over root‐derived resources. As a result, microbes have evolved various mechanisms to kill, damage, suppress, and manipulate their competitors (Granato et al., 2019). Some of the best characterized weapons come from descriptions of *P*.* aeruginosa*—an important rhizosphere microbe—and include poisoned spearguns, protein toxins, mechanical weapons that punch holes in other cells, viruses that kill nonclonemates, and the ability to make molecules like hydrogen cyanide that can damage bacteria (Granato et al., 2019). These alter the composition, distribution, and abundances of microbes with which host crops can then interact. From an evolutionary vantage point, the tactics can also be extremely costly to enact, and potentially divert energy away from mutualistic trade with the host (Engelmoer et al., 2014). Competition among microbes within a host can be directly damaging, and some hosts evolve specific compartments to spatially separate different microbial lineages. Such compartmentalization, in which one‐to‐many interactions can effectively function like one‐to‐one interactions, has allowed hosts to increase the benefits that they obtain from symbiotic partners by allowing the host to direct rewards (or inflict punishments) to specific microbial lineages (Chomicki et al., 2020).

Antibiotics are an important class of compounds that allow a microbe to kill and or disable a competitor, potentially leading to an increase in the microbial actor's relative fitness within a specific environment (Westhoff et al., 2020). In some cases, the release of these compounds provide protection across a physical space, in which multiple plants are growing simultaneously, or across time, in which case future generations of hosts may benefit from what is known as “soil legacy” (Yuan et al., 2018). From an evolutionary perspective, the production of such public goods requires careful examination to determine how these traits evolved, and who they benefit. One of the most well‐studied examples involves *Pseudomonas* bacteria that colonize roots and produce antifungal compounds, such as PHZ‐1‐carboxylate and DAPG, leading to disease‐suppressive soils (Cook et al., 1995; Weller et al., 2002). Presumably, there is a metabolic cost to individual *Pseudomonas* cells in producing antifungal metabolites, and the direct benefits of producing the compounds that build suppressive soils (with diffuse benefits to many potential actors) are not always clear. It is often cited that these compounds can increase the availability of amino acids in root exudates, as well as simultaneously slowing the growth of pathogenic competitors, and thereby indirectly provide a fitness benefit to “a combination of benefits [that] could promote growth of the organisms producing them” (Phillips et al., 2004). However, proving the link between the promotion of growth and the production of the compounds is difficult. One idea is that if root exudates themselves attract pathogens, then plant may benefit from simply having rhizosphere microbes consume the exudates that are acting as a beacon for antagonists (Box 1b) (Denison et al., 2003). Regardless of the mechanism, it is clear that the evolutionary persistence of antifungal warfare strategy by *Pseudomonas* likely contributes to plant health as a by‐product, but the maintenance of this trait is due to the direct benefits for *Pseudomonas* (Granato et al., 2019).

Plants can likewise benefit from interactive networks of competing microbial communities (Durán et al., 2018). The common adage “the enemy of my enemy is a friend” describes numerous competitive interactions in the rhizosphere, but again, this outcome is very context dependent (Table 1). *Rhizoctonia solani* is a soil‐borne fungal pathogen of mung bean seedlings causing root rot. Hosts benefit when the mycoparasite *Trichoderma virens* infects the mycelium of *R*. *solani* to suppress its growth (Inayati et al., 2020). Similarly, the powdery mildew pathogen *Podosphaera plantaginis*, albeit not a soil‐borne pathogen, is subject to frequent attacks by the hyperparasite *Ampelomyces* (Parratt & Laine, 2018). Although infection of *P*. *plantaginis* by the hyperparasite does not protect the host plant from infection, it does significantly reduce pathogen growth and production of its overwintering structures.

While these cases suggest that microbe‐microbe competition consistently facilitate by‐product benefits for hosts, pathogens or commensalists outcompeting mutualists on crop plants are also common (Ma et al., 2014; Snelders et al., 2020). For example, interactions among microbial competitors can lead to host homeostasis, with no net positive or negative effect on the host (Table 1). In these cases, rhizosphere microbes have been shown to *reverse* inhibition of root growth stimulated by other bacteria (*Agrobacterium* and *Arthrobacter*) in the community. Microbes in the bacterial genus *Variovorax* were shown to enforce a chemical homeostasis in plants, such that the host is able maintain its “own” developmental program even when it is embedded in a complex community of microbes (Finkel et al., 2020). The direct benefit to the microbial actor was not investigated, but it was suggested that *Variovorax* relied on bacterially produced substrates to survive, not plant‐derived substrates. This suggests that the microbial actor may have evolved the trait in response to benefits gained by direct interactions with other microbes, rather than benefits provided by the host (Box 1c).

### 3.2 Microbe–microbe facilitation

While competitive interactions play a key role in mediating microbial dominance on a root system, infection by one rhizosphere microbe can facilitate the colonization of another (Table 1) as extensively reviewed in Zélé et al. (2018). The consequences for the host plant are again very context dependent. These interactions can be external, for example, when fungi “trap” phosphate solubilizing bacteria on hyphae to facilitate P uptake (Taktek et al., 2015), or they can be intracellular, whereby one organism facilitates the entry of another into a host cell.

One of the best examples of these intracellular facilitation dynamics is found between AM fungi and *Trichoderma* fungal species. Although both taxa are used to improve plant growth and/or control root pathogens, when they occur together the outcome for the host depends on the specific facilitation dynamics of the interacting host and fungal strains. Plant species in the Brassicaceae family (including important crop plants such as *Brassica napus*, *B*. *oleracea*, and the model organism *A*. *thaliana*) have lost the ability to form symbiosis with AM fungi (Cosme et al., 2018), and the presence of AM fungi can even elicit negative responses in nonhost plants (Lambers & Teste, 2013). However, inoculation of plants with both AM fungi and the fungus *T*. *harzianum* was found to significantly increase the colonization by AM fungi, resulting in an increased productivity in two Brassicaceae species (Poveda et al., 2019). Whether such interactions are stable over subsequent generations is unknown because indirect facilitation dynamics between AM fungi and *Trichoderma* are not consistently beneficial to hosts (Harman et al., 2004). AM fungi have been shown to facilitate the colonization of *Trichoderma*—but with negative effects for the host. So‐called “mycoparasitism” has evolved whereby *T*. *harzianum* penetrates the wall (i.e., spores, hyphae, and intracellular structures) of AM fungi through local hydrolysis of the wall polymers. The authors found that while there were no significant differences in the total number of AM fungal spores when grown in either presence or absence of *Trichoderma*, spores formed in the presence of *Trichoderma* were damaged (De Jaeger et al., 2010). This demonstrates that microbe–microbe facilitation dynamics need to be carefully considered before dual inoculation is recommended to improve crop performance (Lace et al., 2015).

### 3.3 Nested manipulations

Plants can experience benefits and costs as they interact with microbes from the rhizosphere. These microbes themselves can likewise be colonized intracellularly by phylogenetically diverse bacterial endosymbionts that modify microbial uptake and nutrient delivery to hosts. These can drive the evolution of strict and nested interdependencies (Turrini et al., 2018), which ultimately alter both microbial and plant fitness (Ghignone et al., 2012) (Table 1). For example, nested endosymbionts in fungal networks support numerous host functions including type II and type III secretion systems, synthesis of vitamin B12, and even the production of antibiotics and toxin‐resistance molecules (Ghignone et al., 2012).

Compared to free‐living bacteria, nested endosymbionts enjoy a relatively stable environment inside of fungal hosts, free from predation, competition, and grazing. While such endobacteria can provide benefits to their fungal hosts, they also exert significant costs. *Mortierella elongata* is a growth promoting fungus that hosts the bacteria symbiont *Mycoavidus cysteinexigens*. The bacteria consume fungal host‐derived fatty acids, leading to fungal respiration rates ~30% higher compared to fungus lacking the bacterial partner (Uehling et al., 2017). Such costs can be passed on to hosts, even though hosts have no direct way of controlling nested endosymbionts.

Such costly nested symbioses are found across agriculturally important microbes (Alabid et al., 2019) and have the potential to modulate the efficacy of direct benefits conferred to crop hosts by fungal mutualists. A prominent example is the pathogenicity of the fungus, *Rhizopus microsporus*, on rice plants. While historically, the *Rhizopus* fungus was identified as the cause of rice seedling blight, disease symptoms are actually caused by a nested bacterial endophyte: Endophytic *Burkholderia rhizoxinica*, and not the fungal host, produce the antimitotic rhizoxin toxin that causes symptoms of blight (Bastías et al., 2020; Partida‐Martinez & Hertweck, 2007). The bacteria benefit from this nested relationship via increased dispersal with fungal spores, and an increased ability to directly obtain nutrients from the fungal network. The fungus, likewise, gains a rhizoxin toxin weapon to increase its own fitness despite lacking the metabolic capacity to produce such a toxin itself. The evolutionary consequences of nested manipulations to crop productivity require more research (Alabid et al., 2019).

## 4 OUTLOOK

The large variety of ways, in which rhizosphere microbes can directly and indirectly impact plant performance, places them at the center of the next generation of solutions for enhancing plant productivity and resilience. As the examples above illustrate, the view of rhizosphere microbes as passive accessories to plant performance ignores the myriad evolutionary pressures shaping microbial functional ecology. These pressures, combined with indirect effects, and context dependence, can produce a range of outcomes for plant performance, even from rhizosphere microbes that are assumed to be beneficial. We therefore argue that an evolutionary understanding of the ways, in which microbes directly and indirectly manipulate plant fitness will become increasingly important as we attempt to engineer microbial communities for crop production. This *microbe*‐*centric* view acknowledges that for a costly behavior to evolve that benefits a host plant, individuals, and/or clone mates performing that behavior must receive a direct benefit. Building on this insight, a key next step is to develop spatially refined techniques and fitness assays to link specific cooperative behaviors with direct fitness gains for members of microbial consortia. Accounting for the fitness outcomes for microbial partners will allow better prediction of the stability of beneficial plant–microbe interactions. Likewise, experimental evolution has become a powerful tool to study the precise selection pressures driving antagonistic strains to evolve mutualistic behavior (Li, de Jonge, et al., 2021; Li et al., 2021; Manriquez et al., 2021). However, because of the difficulties in following the evolution of traits in genetically diverse consortia over time, these experiments are often performed in low‐diversity (e.g., one strain) systems (Marchetti et al., 2014, 2017; Quides et al., 2021). Therefore, a major new frontier is in following the evolution of microbial behaviors in complex webs of interacting species (Manriquez et al., 2021). This is possible using “evolve and resequence” techniques that rely on estimating relative strain fitness in large synthetic community of naturally variable strains (Burghardt et al., 2018), and mass sequencing that allows researchers to identify genetic mechanisms underlying adaptation in bacteria at the genomic level (Bailey & Bataillon, 2016).

The context dependency of many microbial outcomes (both direct and indirect) likewise increases the difficulty in predicting and engineering these behaviors. Even small changes in conditions such as soil nutrient availability can lead to dramatic switches in a microbial behavior from beneficial to deleterious effects even when mediated by a single bacterial volatile compound (Morcillo et al., 2020). There has been some success with host‐mediated “community selection” of microbes (e.g., Yin et al., 2021). These experiments rely on successive plantings of the same host genotype under a range of conditions. While they can lead to the accumulation of a community of more beneficial microbes, this is not a consistent outcome. Instead, effects might be limited to the accumulation of specific groups of microbes, like disease‐suppressive microbes, that directly benefit from blocking pathogen growth (Weller et al., 2002) (i.e., an indirect effect to plant hosts).

This evolutionary vantage point can also help identify specific microbial traits that can be improved for cropping systems. For example, if microbes are able to provide better (or more) information to hosts about spatial variation in the abiotic and biotic environment (e.g., water, nutrients, and pathogens) and facilitate the spatial distribution of growth to maximize plant performance, this could be evolutionarily robust because the interests of the host and microbe are more closely aligned (Denison, 2019). More generally, advances in integrating microbiomes into crop science are likely to be strongly stimulated by a broader recognition that microbes are under selection to increase their own fitness. To ensure persistent benefits from putatively cooperative microbes, hosts may require specific mechanisms that allow them to identify if (and when) they are being manipulated by external parties, and whether such manipulation is costly or beneficial. Such mechanisms should be a topic for study, and target for crop improvement. When combined with a deeper understanding of rhizosphere community dynamics, this knowledge will help future crops truly harness the diverse benefits of rhizosphere microbes.

## CONFLICT OF INTEREST

The authors declare that they have no conflict of interest.

## Data Availability

Data sharing is not applicable to this article as no new data were created or analyzed in this study.
